# Clinical Epidemiology, Etiology, and Outcomes of Upper Gastrointestinal Bleeding at a Tertiary Center in Bahrain: A Retrospective Study

**DOI:** 10.7759/cureus.77133

**Published:** 2025-01-08

**Authors:** Yousif F Yousif, Mahmood B Dhaif, Ali A Alaysreen, Saad I Mallah, Moosa AlHoda, Husain A Alrahma, Ahmed A Alekri, Tahera H Qaroof, Ahmed Alsaegh

**Affiliations:** 1 Department of Surgery, The Royal Marsden NHS Foundation Trust, London, GBR; 2 Department of Internal Medicine, Salmaniya Medical Complex, Manama, BHR; 3 Department of Family Medicine, Salmaniya Medical Complex, Manama, BHR; 4 Department of Surgery, Barking, Havering and Redbridge University Hospitals NHS Trust, London, GBR; 5 Department of Medicine, RCSI (Royal College of Surgeons in Ireland) - Medical University of Bahrain, Al Sayh, BHR; 6 Department of Gastroenterology and Hepatology, Salmaniya Medical Complex, Manama, BHR; 7 Department of General Practice, RCSI (Royal College of Surgeons in Ireland) - Medical University of Bahrain, Al Sayh, BHR; 8 Department of General Practice, Manama Medical Center, Manama, BHR; 9 Department of Psychiatry, Salmaniya Medical Complex, Manama, BHR

**Keywords:** bahrain, gastric variceal bleeding, non-variceal upper gastrointestinal bleeding, ugib, upper gi bleeds

## Abstract

Background

Upper gastrointestinal bleeding (UGIB) is one of the most common major medical emergencies. This study sought to determine the epidemiology, clinical characteristics, and outcomes of UGIB in the largest major tertiary care center in Bahrain, compared to regional and international cohorts.

Methods

We conducted a retrospective cohort study of all patients diagnosed with UGIB between April 2021 and April 2022 in Salmaniya Medical Complex, Bahrain's largest tertiary-level public hospital. The primary outcomes measured included 30-day mortality rates and one-year readmission rates. Other variables collected included demographic factors, baseline characteristics, comorbidities, symptomatology, endoscopic findings, and etiologies of the bleeding.

Results

A total of 212 patients with UGIB were included. The mean age of the patients was 56.7 ± 19.1 years. More than 50% of patients with UGIB presented with melena and symptoms of anemia. The most common cause of UGIB in Bahrain was duodenal ulcers, which were found in 75 patients (37.7%). One in two patients with UGIB required packed red blood cells, while fresh-frozen plasma and platelet transfusions were reserved for severe cases. The readmission rate within one year of discharge (14.2%) was associated with smoking, cardiac history, melena, gastric malignancy, and rescope during admission. The 30-day mortality (15.6%) was associated with comorbidities of chronic kidney disease, cerebrovascular disease, and hematochezia on presentation.

Conclusion

Overall, the mortality rate of UGIB in Bahrain is higher than in countries in the region, the UK, and the US, signaling potential gaps in management and a reflection of a more complex patient population.

## Introduction

Upper gastrointestinal bleeding (UGIB) is one of the most common major medical emergencies [1]. It is estimated that UGIB contributes to up to 150 hospital admissions per 100,000 adults worldwide [1]. Anatomically, bleeding from the gastrointestinal tract above the Treitz ligament level is considered a UGIB. UGIB is generally classified according to cause as variceal UGIB (VUGIB) or non-variceal UGIB (NVUGIB). The estimated mortality rates range between 2% and 15% [1,2]. Multiple factors are associated with mortality outcomes, including the cause of UGIB, the presence of liver disease, cardiovascular comorbidity, the need for blood and blood product transfusions, the use of anticoagulants, and various laboratory parameters [3-5]. 

UGIBs have a wide variety of clinical presentations, including coffee-ground emesis, hematemesis, hematochezia, melena, abdominal pain, and hemodynamic instability secondary to hemorrhagic shock (presenting as tachycardia, hypotension, and altered mental status). The clinical manifestations, severity, and overall prognosis of the UGIB patient vary according to the etiology and comorbidities [6]. Therefore, to enhance patient outcomes and reduce the risk of complications, a prompt and accurate diagnostic and therapeutic strategy is required [7].

Multiple approaches are needed to salvage the UGIB patient. Multi-stage management is recommended, including pre-endoscopic management (hospitalization and blood transfusion), endoscopic management (thermocoagulation and sclerosant injection within 24 hours), and pharmacologic management (injection of proton pump inhibitors, PPIs) [8]. Esophagogastroduodenoscopy (EGD) is an imperative diagnostic and therapeutic intervention that identifies the site of bleeding and causative pathology, with the opportunity to obtain histological evidence [2].

The epidemiology and clinical course of UGIB vary significantly across countries and regions. The mortality rate in the US and the UK is estimated to be between 5% and 10% [9]. The most common cause of UGIB in the US is peptic ulcer disease (PUD). Peptic ulcers affect up to 40% of adults in the US, with duodenal ulcers appearing more common than gastric ulcers [7,10]. On the contrary, a recent review of an Egyptian cohort found UGIB to be mainly secondary to variceal bleeding; this is related to the high incidence rate of hepatitis C in Egypt. In cases of liver cirrhosis, the estimated mortality rate was 20% at six weeks from hospital admission [11]. Beyond blood loss, UGIB may have catastrophic effects on the liver, leading to hepatic coma and death in chronic cases [12,13].

In the Arabian Gulf countries, a recent study in Saudi Arabia, including 259 patients, reported that the cause of UGIB in their population was predominantly non-variceal bleeding (80.1%), and the in-hospital mortality rate was reported to be 4.4% [14]. As for Bahrain, the only retrievable study was conducted in 1998 on 186 patients, reporting a direct mortality rate (attributable to bleeding) of 11.2%. Duodenal ulcers were responsible for 87 (53%) of cases, followed by 17 cases (10.2%) of esophageal varices and 11 cases (6.6%) of gastric ulcers. Blood transfusions were required in 126 patients (67%) [15].

This study aims to determine the epidemiology, clinical characteristics, readmission, and 30-day mortality outcomes, and their predictors in the largest public tertiary hospital in Bahrain. We will also compare the characteristics of our population to regional and international cohorts to better understand any factors that may be uniquely inherent to this population. This study will thus inform our public health and clinical strategy in approaching UGIB nationally, in Bahrain, and regionally, in the wider Gulf and Middle East.

## Materials and methods

Study design

A retrospective cohort study of all patients diagnosed with UGIB between April 2021 and April 2022 in Salmaniya Medical Complex (SMC) - the largest (1200-bed) tertiary-level public hospital in Manama, Bahrain, with its Emergency Department (ED) alone receiving over 300,000 patients annually. SMC is the only government-funded public tertiary hospital in Bahrain, accessible to the entire population of the Kingdom, which includes approximately 1.57 million Bahraini and non-Bahraini residents [16].

Inclusion and exclusion criteria

All patients who underwent EGD for UGIB between April 1, 2021, and April 1, 2022, have been included. All patients for whom an EGD was done for other indications, and those for whom UGIB was later ruled out, were excluded from the study.

Initially, 270 patients suspected of UGIB and with an EGD procedure on file at SMC within the period were screened. The National Identification Numbers were retrieved via the Logbook Record from the Endoscopy Unit at SMC, and endoscopy findings were reviewed on the electronic database (I-SEHA, the Integrated System for Electronic Health Records). Of the 270 patients with suspected UGIB, 223 were confirmed via EGD, while the procedure ruled out the diagnosis in 47 patients.

Data collection

Additional data retrieved from the electronic database (I-SEHA) include demographics (e.g., age, sex, nationality), health behaviors (e.g., smoking status, alcohol consumption), comorbidities (e.g., diabetes mellitus), medications (e.g., non-steroidal anti-inflammatory drugs (NSAIDs), warfarin), medical history (e.g., previous upper or lower gastrointestinal bleeding, previous PUD, varices), clinical features on presentation, endoscopic findings, type of intervention (if any), and physical health status (heart rate, blood pressure, oxygen saturation). Additionally, the following laboratory values were collected: hemoglobin level, platelet count, the international normalized ratio (INR), alanine aminotransferase (ALT), alkaline phosphatase (ALP), and serum creatinine. In terms of imaging, endoscopic findings were recorded. In terms of clinical course and outcomes, these included any blood and blood-related product transfusions (e.g., whole blood, packed red blood cells (PRBCs), fresh frozen plasma (FFP)), related surgery, 30-day mortality related to UGIB, and one-year readmission.

Definitions

For definitions of the terms and criteria used in our study, please refer to Table 1.

**Table 1:** Definitions of the terms and criteria used in the study.

Term/criteria	Definition
Charlson Comorbidity Index (CCI)	The Charlson Comorbidity Index (CCI) is a widely used method for categorizing comorbidities of patients based on the International Classification of Diseases (ICD) diagnosis codes. Each comorbidity category has an associated weight, which is determined by the adjusted risk of mortality or resource use [17].
Cardiac history	Cardiac history refers to the presence of conditions such as myocardial infarction and congestive heart failure. Myocardial infarction indicates a history of a heart attack, clinically documented with supporting diagnostic evidence. Congestive heart failure is characterized by symptoms such as shortness of breath, fatigue, and fluid retention, confirmed through clinical documentation.
Peripheral vascular disease	Peripheral vascular disease involves vascular issues affecting the peripheral arteries, typically identified through clinical evidence like intermittent claudication or peripheral artery disease. Cerebrovascular disease encompasses conditions such as stroke or transient ischemic attack (TIA), supported by documentation of neurological deficits or imaging studies revealing previous cerebrovascular events.
Dementia	Dementia refers to cognitive impairment, confirmed through clinical assessments and cognitive testing. Chronic obstructive pulmonary disease (COPD) includes chronic lung diseases such as chronic bronchitis or emphysema, documented via clinical evaluation and pulmonary function tests.
Rheumatologic disease	Rheumatologic disease covers autoimmune or inflammatory conditions like rheumatoid arthritis or systemic lupus erythematosus, diagnosed through clinical assessment and relevant diagnostic tests. Peptic ulcer disease involves a history of gastric or duodenal ulcers, confirmed by clinical evaluation and endoscopic findings.
Liver disease	Liver disease includes chronic conditions such as cirrhosis or chronic hepatitis, supported by clinical and laboratory documentation. Diabetes mellitus is categorized into uncomplicated diabetes, where no complications like neuropathy or nephropathy are present, and complicated diabetes, which involves complications such as diabetic retinopathy or nephropathy.
Renal disease	Renal disease is divided into two categories: mild to moderate renal disease, which corresponds to chronic kidney disease stages 3-4 with a glomerular filtration rate (GFR) between 30 and 59 mL/min/1.73 m², and severe renal disease, which includes chronic kidney disease stage 5 or end-stage renal disease (ESRD), defined by a GFR less than 30 mL/min/1.73 m² or the need for dialysis.
Cancer	Refers to the presence of any form of cancer, whether active or with a history of the disease, documented by clinical evaluation and diagnostic tests. Metastatic cancer describes cancer that has spread beyond its original site, confirmed by imaging studies or clinical findings.
One-year readmission	Refers to the readmission of patients within one year of their initial upper gastrointestinal bleeding (UGIB) episode. This readmission is specifically related to the recurrence of UGIB rather than the impact of comorbidities.

Endoscopy protocol in Bahrain

Pre-endoscopic Risk Categorization

In Bahrain, similar to the UK, patients presenting with UGIB are categorized based on pre-endoscopic risk scores to guide management and determine the urgency of endoscopy [18,19]. Commonly used risk stratification tools include the Glasgow-Blatchford score (GBS) and the Rockall score.

Glasgow-Blatchford score (GBS): This score assesses the risk of requiring medical intervention or death before endoscopy. It includes parameters such as blood urea nitrogen, hemoglobin levels, systolic blood pressure, pulse rate, presence of melena, syncope, hepatic disease, and cardiac failure. A low GBS (score, 0-1) identifies patients at low risk who can be managed as outpatients.

Rockall score: This score is used post-endoscopy, but includes a pre-endoscopic component to predict mortality. It considers age, shock (systolic blood pressure and pulse rate), comorbidity, and initial endoscopic findings.

Timing of Endoscopy

The timing of endoscopy is crucial in managing UGIB. According to the European Society of Gastrointestinal Endoscopy (ESGE) guidelines [20], which are also followed in Bahrain.

Urgent endoscopy: This endoscopy is performed within 12 hours for patients with high-risk features, such as hemodynamic instability (e.g., hemorrhagic shock), after initial resuscitation and stabilization.

Early endoscopy: This endoscopy is conducted within 24 hours of presentation for patients without hemodynamic instability. This timing helps in diagnosing and managing the source of bleeding, thus reducing the risk of further bleeding and other complications.

Endoscopic Risk Classification

Upon endoscopy, bleeding lesions are classified using the Forrest classification system, which predicts the risk of rebleeding and guides further therapeutic decisions. Forrest I: active bleeding (spurting or oozing); Forrest II: recent bleeding with stigmata such as visible vessels or adherent clots; Forrest III: lesions without active bleeding (clean-based ulcers).

Therapeutic Interventions

Based on the findings, endoscopic hemostatic techniques, such as injection therapy, thermal coagulation, or mechanical hemostasis, are employed. Patients are also managed with PPIs pre- and post-endoscopy to reduce rebleeding risk and improve outcomes.

Criteria for Re-endoscopy

The criteria for performing second-look endoscopies (re-endoscopies) at SMC are based on guidelines from the ESGE and the American College of Gastroenterology. Routine second-look endoscopy is generally not recommended. However, it is performed in patients with high-risk stigmata, such as a visible vessel or active bleeding noted on initial endoscopy, or in cases where there are clinical signs of re-bleeding. These guidelines ensure that only patients with the highest risk of complications are subjected to this procedure [20,21].

Statistical analysis

Statistical analysis was performed using IBM SPSS Statistics for Windows, Version 28 (Released 2021; IBM Corp., Armonk, NY, USA). Quantitative data were presented as means, standard deviations (SDs), and interquartile ranges (IQRs), and analyzed for significance via an unpaired Student's t-test. Categorical data were presented as frequencies and percentages, while continuous variables were expressed as means and SDs. Specific parameters were further investigated for their association with one-year readmission and 30-day mortality related to UGIB. For bivariate analysis, comparisons between categorical variables were conducted using Pearson’s Chi-square test or Fisher’s exact test, as appropriate. Continuous variables were analyzed using an independent samples t-test, while the Mann-Whitney U test was used for p-value calculation with non-normally distributed data. In addition, binary logistic regression was performed to identify and assess potential risk factors for one-year readmission and 30-day mortality, with odds ratios (ORs) and 95% confidence intervals (CIs) reported for significant associations.

Ethics

The study obtained ethical approval from the Research Committee for Government Hospitals before initiating data collection (research approval serial number: 85180723). To reduce the likelihood of human error, we used automated tools to extract the data from electronic patient charts and then transferred the data to SPSS version 28 for verification and analysis. The information was then duplicated digitally in case of data loss. Patients' names and national identification numbers were replaced with randomized IDs to protect their anonymity and privacy.

## Results

A total of 212 patients with UGIB were included in this study. The demographic characteristics of patients presenting with UGIB in Bahrain are detailed in Table 2. The majority were male (68.4%), with a mean age of 56.7 ± 19.1 years. The age group with the highest representation was between 61 and 75 years (29.2%). Most patients (94.8%) presented through the ED, with only 5.2% being inpatients at the time of the UGIB event. Analysis of associations between sociodemographic characteristics and outcomes revealed that non-smokers and non-alcohol consumers had a higher 30-day mortality rate (18.5%, p = 0.029) and (17.4%, p = 0.049), respectively. It is worth noting, however, that this is likely due to the small population of smokers (12 patients) and alcohol consumers (three patients) in our population, and confounded by other variables, such as the etiology of the UGIB.

**Table 2:** Distribution of sociodemographic characteristics and associations between demographics characteristics and the occurrence of readmission within 1-year and 30-day mortality. *Significant at 0.05; **Significant at 0.01 p-values were computed by the Chi-square test or Fisher’s exact test.

Sociodemographic characteristics	n (%)	Readmission, n (%)	Mortality, n (%)
				Age			
≤30 years	25 (11.8)	3 (12)	0 (0)
31-45 years	41 (19.3)	5 (12.2)	2 (4.9)
46-60 years	49 (23.1)	11 (22.4)	4 (8.2)
61-75 years	62 (29.2)	5 (8.1)	15 (24.2)
>75 years	35 (16.5)	6 (17.6)	12 (34.3)
p-value		0.266	<0.001**
Gender (total = 212)			
Male	145 (68.4)	23 (15.9)	19 (13.1)
Female	67 (31.6)	7 (10.6)	14 (20.9)
p-value		0.311	0.146
Nationality (total = 212)			
Bahraini	164 (77.4)	27 (16.6)	29 (17.7)
Non-Bahraini	48 (22.6)	3 (6.3)	4 (8.3)
p-value		0.072	0.116
Department (total = 212)			
Emergency Department	201 (94.8)	29 (14.5)	30 (14.9)
Inpatient	11 (5.2)	1 (9.1)	3 (27.3)
p-value		1	0.383
Smoking status (total = 210)			
Smoker	30 (14.3)	10 (23.8)	2 (4.8)
Ex-smoker	12 (5.7)	-	-
Non-smoker	168 (80)	20 (12)	31 (18.5)
p-value		0.051	0.029*
Drinking alcohol (total = 210)			
Yes	20 (9.5)	3 (15)	0 (0)
No	190 (90.5)	27 (14.3)	33 (17.4)
p-value		1	0.049*

In terms of comorbidities, our population carries a high burden of cardiovascular risk factors, such as hypertension (47.2%) and diabetes (38.7%) - many of whom (18.4%) also suffer from end-organ damage, including a history of cardiac disease (24.5%), among others detailed in Table 3. Several comorbidities were significantly associated with increased 30-day mortality. These included diabetes (25.6%, p = 0.001) - especially with organ damage (30.8%, p = 0.004) - as well as hypertension (29%, p < 0.001), positive cardiac history (32.7%, p < 0.001), specifically heart failure (50%, p = 0.002), and a history of prior myocardial infarction (MI) (37.8%, p < 0.001). The presence of cerebrovascular accident (70%, p < 0.001), especially with hemiplegia (66.7%, p < 0.001), was similarly associated with increased 30-day mortality. The mean Charlson Comorbidity Index (CCI) score for the cohort was 3 ± 3. We found that a higher CCI score was significantly associated with 30-day mortality. Among the patients who died within 30 days, the median CCI score was 6 (IQR: 2), p < 0.001 (Table 4). We documented the possible medications that would increase the risk for UGIB, such as antiplatelet agents, NSAIDs, anticoagulation, and oral steroids. The use of these medications in our population is outlined in detail in Table 5. Among the medications, warfarin use was strongly associated with increased 30-day mortality (50%, p = 0.003), though no association was found with one-year readmission.

**Table 3:** Distribution of comorbidities and associations between comorbidities and the occurrence of readmission within 1-year and 30-day mortality. *Significant at 0.05; **Significant at 0.01 p-values were computed by the Chi-square test or Fisher’s exact test.

Comorbidities	Yes, n (%)	No, n (%)	Total, n (%)	Yes/no	Readmission, n (%)	p-value	Mortality, n (%)	p-value
									Diabetes	82 (38.7)	130 (61.3)	212 (100)	Yes	13 (16)	0.548	21 (25.6)	0.001**
No	17 (13.1)	12 (9.2)															
Uncomplicated	40 (18.9)	172 (81.1)	212 (100)	Yes	6 (15)	0.875	8 (20)	0.39
				No	24 (14)		25 (14.5)	
Organ damage	39 (18.4)	173 (81.6)	212 (100)	Yes	5 (13.2)	0.836	12 (30.8)	0.004**
				No	25 (14.5)		21 (12.1)	
Hypertension	100 (47.2)	112 (52.8)	212 (100)	Yes	12 (12.1)	0.412	29 (29)	<0.001**
				No	18 (16.1)		4 (3.6)	
Cardiac history	52 (24.5)	160 (75.5)	212 (100)	Yes	8 (15.7)	0.73	17 (32.7)	<0.001**
				No	22 (13.8)		16 (10)	
Prior myocardial infarction	37 (17.5)	174 (82.5)	211 (100)	Yes	6 (16.7)	0.654	14 (37.8)	<0.001**
				No	24 (13.8)		19 (10.9)	
Heart failure	14 (6.7)	195 (93.3)	209 (100)	Yes	2 (14.3)	1	7 (50)	0.002**
				No	26 (13.4)		26 (13.3)	
Peripheral vascular disease	2 (0.9)	210 (99.1)	212 (100)	Yes	1 (50)	0.265	2 (100)	0.024*
				No	29 (13.9)		31 (14.8)	
Cerebrovascular accident	10 (4.7)	202 (95.3)	212 (100)	Yes	0 (0)	0.364	7 (70)	<0.001**
				No	30 (14.9)		26 (12.9)	
Hemiplegia	9 (4.3)	202 (95.7)	211 (100)	Yes	0 (0)	0.615	6 (66.7)	<0.001**
				No	29 (14.4)		26 (12.9)	
Chronic liver disease	15 (7.1)	197 (92.9)	212 (100)	Yes	4 (26.7)	0.238	2 (13.3)	1
				No	26 (13.3)		31 (15.7)	
Mild	4 (1.9)	207 (98.1)	211 (100)	Yes	1 (25)	0.463	1 (25)	0.485
				No	29 (14.1)		31 (15)	
Moderate/severe	10 (4.8)	199 (95.2)	209 (100)	Yes	3 (30)	0.161	1 (10)	1
				No	27 (13.6)		30 (15.1)	
Hepatitis C	9 (4.2)	203 (95.8)	212 (100)	Yes	3 (33.3)	0.12	2 (22.2)	0.633
				No	27 (13.4)		31 (15.3)	
Malignancy	12 (5.7)	200 (94.3)	212 (100)	Yes	3 (25)	0.384	2 (16.7)	1
				No	27 (13.6)		31 (15.5)	
No metastasis	7 (3.3)	204 (96.7)	211 (100)	Yes	2 (28.6)	0.263	1 (14.3)	1
				No	28 (13.8)		32 (15.7)	
Metastatic	5 (2.4)	206 (97.6)	211 (100)	Yes	1 (20)	0.541	1 (20)	0.577
				No	29 (14.1)		32 (15.5)	
Hematological malignancy	0 (0)	212 (100)	212 (100)	Yes	0 (0)	Not computed	0 (0)	Not computed
				No	30 (14.2)		33 (15.6)	
Chronic kidney disease	29 (13.7)	183 (86.3)	212 (100)	Yes	2 (6.9)	0.388	8 (27.6)	0.093
				No	28 (15.4)		25 (13.7)	
Moderate/severe	14 (6.6)	198 (93.4)	212 (100)	Yes	0 (0)	0.228	5 (35.7)	0.048*
				No	30 (15.2)		28 (14.1)	
Chronic obstructive pulmonary disease	2 (0.9)	210 (99.1)	212 (100)	Yes	0 (0)	1	0 (0)	1
				No	30 (14.4)		33 (15.7)	
Dementia	0 (0)	212 (100)	212 (100)	Yes	0 (0)	Not computed	0 (0)	Not computed
				No	30 (14.2)		33 (15.6)	
Connective tissue disease	0 (0)	212 (100)	212 (100)	Yes	0 (0)	Not computed	0 (0)	Not computed

**Table 4:** Difference in median of CCI score according to the occurrence of readmission within 1-year and 30-day mortality. **Significant at 0.01 p-values were calculated by using the Mann-Whitney test. IQR, Interquartile range; CCI, Charlson Comorbidity Index

Readmission/mortality	CCI	p-value
	Median (IQR)	
Readmission		
Yes	2.5 (4)	0.735
No	3 (4)	
Mortality		
Yes	6 (2)	<0.001**
No	2 (4)	

**Table 5:** Distribution of medications and distribution of GI PMH and associations between medications/GI PMH and the occurrence of readmission within 1-year and 30-day mortality. **Significant at 0.01 p-values were computed by the Chi-square test or Fisher’s exact test. PUD, Peptic ulcer disease; GI PMH, Gastroenterology past medical history

Medications/GI PMH	Yes, n (%)	No, n (%)	Yes/no	Re-admission, n (%)	p-value	Mortality, n (%)	p-value
								Medications							
Non-steroidal anti-inflammatory drugs	14 (6.7)	196 (93.3)	Yes	3 (21.4)	0.428	1 (7.1)	0.698
			No	27 (13.8)		30 (15.3)	
Aspirin	37 (17.6)	173 (82.4)	Yes	4 (10.8)	0.506	8 (21.6)	0.195
			No	26 (15)		23 (13.3)	
Clopidogrel	10 (4.8)	200 (95.2)	Yes	2 (20)	0.638	3 (30)	0.169
			No	28 (14)		28 (14)	
Novel oral anticoagulants	9 (4.3)	201 (95.7)	Yes	3 (33.3)	0.122	3 (33.3)	0.132
			No	27 (13.4)		28 (13.9)	
Warfarin	12 (5.7)	198 (94.3)	Yes	0 (0)	0.223	6 (50)	0.003**
			No	30 (15.2)		25 (12.6)	
Low molecular weight heparin	2 (1)	208 (99)	Yes	0 (0)	1	0 (0)	1
			No	30 (14.4)		31 (14.9)	
Steroids	3 (1.4)	207 (98.6)	Yes	0 (0)	1	2 (66.7)	0.058
			No	30 (14.5)		29 (14)	
GI PMH							
Upper or lower gastrointestinal bleed	27 (12.7)	185 (87.3)	Yes	2 (7.7)	0.547	2 (7.4)	0.267
			No	28 (15.1)		31 (16.8)	
PUD	15 (7.1)	197 (92.9)	Yes	2 (13.3)	1	0 (0)	0.135
			No	28 (14.3)		33 (16.8)	
Varices	11 (5.2)	201 (94.8)	Yes	2 (18.2)	0.658	0 (0)	0.22
			No	28 (14)		33 (16.4)	

The most common symptom at the time of presentation was melena (62.1%), followed by anemia (51.7%). Abdominal pain on presentation, which was reported in 37% of patients, was significantly associated with lower 30-day mortality (21.1%, p = 0.002), as was the case with non-bloody vomiting (19.1%, p = 0.031) (Table 6).

**Table 6:** Distribution of clinical features and associations between clinical features and the occurrence of readmission within 1-year and 30-day mortality. *Significant at 0.05; **Significant at 0.01 p-values were computed by the Chi-square test or Fisher’s exact test.

Clinical features	Yes, n (%)	No, n (%)	Yes/no	Readmission, n (%)	p-value	Mortality, n (%)	p-value
								Hematemesis	50 (23.7)	161 (76.3)	Yes	8 (16)	0.68	7 (14)	0.792
No	22 (13.7)	25 (15.5)													
Melena	131 (62.1)	80 (37.9)	Yes	23 (17.6)	0.076	21 (16)	0.654
			No	7 (8.8)		11 (13.8)	
Hematochezia	16 (7.6)	195 (92.4)	Yes	3 (18.8)	0.707	3 (18.8)	0.716
			No	27 (13.8)		29 (14.9)	
Abdominal pain	78 (37)	133 (63)	Yes	14 (17.9)	0.235	4 (5.1)	0.002**
			No	16 (12)		28 (21.1)	
Vomiting	75 (35.5)	136 (64.5)	Yes	11 (14.7)	0.89	6 (8)	0.031*
			No	19 (14)		26 (19.1)	
Coffee-ground	55 (26.1)	156 (73.9)	Yes	8 (14.5)	0.936	8 (14.5)	0.881
			No	22 (14.1)		24 (15.4)	
Anemia	109 (51.7)	102 (48.3)	Yes	15 (13.8)	0.844	18 (16.5)	0.573
			No	15 (14.7)		14 (13.7)	

PUD (confirmed by endoscopic findings) was found to be the most common cause of UGIB (42.5%). Duodenal ulcers were the most common endoscopic finding, seen in 37.7% of all patients. Forrest classifications 2B, 2C, and 1A were associated with higher one-year readmission rates (55.7%, 33.3%, and 30%, respectively, p = 0.04) (Table 7). Testing for *Helicobacter pylori* was conducted in 52 patients, with 36.5% testing positive. Among those positive, 43.5% received eradication therapy. Additionally, 21.7% of patients using NSAIDs or aspirin received PPIs prophylactically (Table 8). Varices were found to be the cause of UGIB in 17.1% of our population. With respect to the etiology of UGIB, only those with gastric cancer had a significantly higher rate of one-year readmission (100%, p = 0.022). No specific etiology was associated with an increased risk of 30-day mortality. The detailed endoscopy findings are outlined in Table 9.

**Table 7:** Distribution of Forrest class and intervention.

Forrest class/intervention	n (%)
Forrest class (total = 86)	
1A	4 (4.7)
1B	10 (11.6)
2A	11 (12.8)
2B	3 (3.5)
2C	3 (3.5)
3	55 (64)
Intervention (total = 35)	
Clip placement	32 (91.4)
Variceal banding	1 (2.9)
Argon plasma coagulation	2 (5.7)

**Table 8:** Distribution of Helicobacter pylori and PPI prophylaxis and associations between Helicobacter pylori/PPI prophylaxis and the occurrence of readmission within 1-year and 30-day mortality. p-values were computed by the Chi-square test or Fisher’s exact test. PPI, Proton pump inhibitor; NSAID, Non-steroidal anti-inflammatory drug

*Helicobacter pylori*/PPI prophylaxis	Yes, n (%)	No, n (%)	Total	Yes/no	Readmission, n (%)	p-value	Mortality, n (%)	p-value
									Helicobacter pylori								
Testing	19 (36.5)	33 (63.5)	52 (100)	Yes	3 (15.8)	0.726	2 (10.5)	0.617
				No	8 (24.2)		2 (6.1)	
Eradication Rx	10 (43.5)	13 (56.5)	23 (100)	Yes	2 (20)	1	0 (0)	0.229
				No	3 (23.1)		3 (23.1)	
PPI prophylaxis								
NSAID/aspirin	46 (21.7)	166 (78.3)	212 (100)	Yes	7 (15.2)	0.826	9 (19.6)	0.398
				No	23 (13.9)		24 (14.5)	
PPI prophylaxis	10 (4.7)	202 (95.3)	212 (100)	Yes	2 (20)	0.637	2 (20)	0.657
				No	28 (13.9)		31 (15.3)	

**Table 9:** Distribution of etiology/endoscopy findings and associations between etiology/endoscopy findings and the occurrence of readmission within 1-year and 30-day mortality. *Significant at 0.05; **Significant at 0.01 p-values were computed by the Chi-square test or Fisher’s exact test. GERD, Gastroesophageal reflux disease

Etiology/endoscopy findings	Yes, n (%)	No, n (%)	Total	Yes/no	Readmission, n (%)	p-value	Mortality, n (%)	p-value
									Inflammatory/erosive								
GERD	20 (10.1)	179 (89.9)	199 (100)	Yes	6 (30)	0.09	3 (15)	0.739
				No	24 (13.4)		24 (13.4)	
Eso/gastritis/duodenitis	38 (19.1)	161 (80.9)	199 (100)	Yes	7 (18.4)	0.522	8 (21.1)	0.134
				No	23 (14.3)		19 (11.8)	
PUD	90 (42.5)	122 (57.5)	212 (100)	Yes	14 (15.6)	0.631	13 (14.4)	0.699
				No	16 (13.2)		20 (16.4)	
Duodenal ulcer	75 (37.7)	124 (62.3)	199 (100)	Yes	10 (13.3)	0.593	9 (12)	0.615
				No	20 (16.1)		18 (14.5)	
Gastric ulcer	27 (13.6)	172 (86.4)	199 (100)	Yes	6 (22.2)	0.257	5 (18.5)	0.379
				No	24 (14)		22 (12.8)	
Varices								
Esophageal	20 (10.1)	179 (89.9)	199 (100)	Yes	5 (25)	0.194	0 (0)	0.082
				No	25 (14)		27 (15.1)	
Fundal	8 (4)	191 (96)	199 (100)	Yes	1 (12.5)	1	1 (12.5)	1
				No	29 (15.2)		26 (13.6)	
Both	6 (3)	193 (97)	199 (100)	Yes	2 (33.3)	0.224	0 (0)	1
				No	28 (14.5)		27 (14)	
Malignancy								
Gastric	2 (1)	197 (99)	199 (100)	Yes	2 (100)	0.022*	1 (50)	0.254
				No	28 (14.2)		26 (13.2)	
Esophageal	2 (1)	197 (99)	199 (100)	Yes	0 (0)	1	1 (50)	0.254
				No	30 (15.2)		26 (13.2)	
Pancreatic	0 (0)	199 (100)	199 (100)	Yes	0 (0)	Not computed	0 (0)	Not computed
				No	30 (15.1)		27 (13.6)	
Traumatic								
Trauma	2 (1)	197 (99)	199 (100)	Yes	0 (0)	1	0 (0)	1
				No	30 (15.2)		27 (13.7)	
Mallory-Weiss	4 (2)	195 (98)	199 (100)	Yes	0 (0)	1	0 (0)	1
				No	30 (15.4)		27 (13.8)	
No finding	33 (16.6)	166 (83.4)	199 (100)	Yes	0 (0)	0.006**	4 (12.1)	1
				No	30 (18.1)		23 (13.9)	

Among laboratory parameters, higher levels of serum creatinine were significantly associated with increased 30-day mortality (median: 104 mmol/L, IQR: 95 mmol/L, p < 0.001). Elevated ALP levels also showed a significant association with 30-day mortality (median: 93 IU/L, IQR: 74.5 IU/L, p = 0.001). Additionally, patients with a higher INR were more likely to experience 30-day mortality (median: 1.23, IQR: 0.36, p < 0.001) (Table 10). Further details regarding vital signs on admission or start of UGIB for inpatients, as well as hematological, hepatic, and renal parameters, are presented in Table 10.

**Table 10:** Descriptive statistics of vital signs (at the time of presentation) and laboratory findings and difference in median of vital signs (at the time of presentation)/laboratory findings according to the occurrence of readmission within 1-year and 30-day mortality. **Significant at 0.01 p-values were calculated by using the Mann-Whitney test. IQR, Interquartile range; HR, Heart rate; SBP, Systolic blood pressure; DBP, Diastolic blood pressure; O_2_ Sat, Oxygen Saturation; INR, International normalized ratio; ALT, Alanine aminotransferase (IU/L); ALP, Alkaline phosphatase (IU/L)

Vital signs/laboratory findings	Total	Mean ± SD	Median (IQR)	Readmission		p-value	Mortality		p-value
				Yes, median (IQR)	No, median (IQR)		Yes, median (IQR)	No, median (IQR)	
										Vital signs									
HR	207	94 ± 58	88 (22)	88 (22)	88 (22)	0.763	84 (28)	88 (22)	0.548
SBP	208	123 ± 22	120 (29)	118 (17)	122 (31)	0.307	120 (26)	120 (30)	0.973
DBP	208	72 ± 14	70 (18)	67 (18)	71 (18)	0.411	70 (20)	71 (18)	0.666
O_2_ Sat	202	98 ± 3	99 (2)	99 (2)	99 (2)	0.507	98 (4)	99 (2)	0.174
Laboratory findings									
Hemoglobin	211	9.6 ± 3.1	9.4 (4.5)	9.4 (4.5)	9.4 (4.5)	0.963	9.3 (3.4)	9.5 (4.8)	0.201
Platelets	211	259 ±136	228 (154)	234 (179)	228 (138)	0.816	259.5 (181)	228 (152)	0.975
INR	205	1.74 ± 6.75	1.10 (0.23)	1.11 (0.32)	1.09 (0.23)	0.532	1.23 (0.36)	1.07 (0.19)	<0.001**
ALT	207	30 ± 37	20 (21)	21 (19)	19 (21)	0.316	19.5 (19.5)	20 (21)	0.98
ALP	207	98 ± 91	78 (42)	85 (35)	78 (42)	0.703	93 (74.5)	77 (42)	0.001**
Creatinine	206	129 ± 182	75 (61)	73 (43)	75 (64)	0.645	104 (95)	71 (54)	<0.001**

We studied the therapeutic interventions used in our hospital for patients with UGIB. Approximately one in two patients (44.1%) received at least one unit of PRBCs, while a small percentage required other blood products. Additionally, 17.5% of patients underwent a second endoscopy during their admission, while 3.3% required surgical intervention (Table 11). Patients who required a second endoscopy (rescope) during admission had a higher one-year readmission rate, with 45.9% of these patients readmitted within a year (p < 0.001). Due to the small sample size of patients who underwent endoscopic interventions, we could not perform a sub-group analysis that would realistically estimate the impact of these interventions on 30-day mortality and one-year readmission (Table 12).

**Table 11:** Distribution of interventions. **Significant at 0.01 p-values were computed by the Chi-square test or Fisher’s exact test. PRBCs, Packed red blood cells; FFP, Fresh frozen plasma; PLT, Platelets and associations between interventions and the occurrence of readmission within 1-year and 30-day mortality

Interventions	Yes, n (%)	No, n (%)	Yes/no	Readmission, n (%)	p-value	Mortality, n (%)	p-value
								Transfusion	PRBC: 93 (44.1), PRBC + FFP: 3 (1.4), PRBC + FFP + PLT: 1 (0.5)	114 (54)	Yes	15 (15.5)	0.633	16 (16.5)	0.62
No	15 (13.2)	16 (14)													
Rescope	37 (17.5)	174 (82.5)	Yes	17 (45.9)	<0.001**	6 (16.2)	0.844
			No	13 (7.5)		26 (14.9)	
Surgery	7 (3.3)	204 (96.7)	Yes	2 (28.6)	0.261	2 (28.6)	0.287
			No	28 (13.7)		30 (14.7)	

**Table 12:** Distribution of outcomes.

Outcomes	Yes	No	Total
	n (%)	n (%)	n (%)
Readmission within 1 year	30 (14.2)	181 (85.8)	211 (100)
30-days mortality	33 (15.6)	179 (84.4)	212 (100)

Binary logistic regression

In the binary logistic regression analysis, smokers were approximately two times more likely to be readmitted within one year (p = 0.033, OR: 3.27, 95% CI: 1.10-9.76). For 30-day mortality, several factors were found to be significantly associated with increased risk. Smokers were 35 times more likely to experience mortality within 30 days, with an OR of 0.028 (95% CI: 0-0.73, p = 0.032). In patients with diabetes, organ damage was strongly associated with increased mortality (OR: 0.002, 95% CI: 0-0.13; p = 0.003). Hypertension also contributed to a significantly higher risk of 30-day mortality (OR: 3068.08, 95% CI: 4.45-2114605.78; p = 0.016). A history of prior MI was another significant risk factor for mortality (OR: 849.69, 95% CI: 6.76-106786.03; p = 0.006). Patients with moderate to severe chronic kidney disease (CKD) also had a markedly increased risk of 30-day mortality (OR: 320455.52, 95% CI: 36.04-2849167704.23; p = 0.006). Laboratory parameters also showed significant associations, with elevated ALP levels being linked to 30-day mortality (OR: 1.057, 95% CI: 1.02-1.09; p = 0.001), as well as lower creatinine levels (OR: 0.958, 95% CI: 0.93-0.99; p = 0.008). Finally, with respect to the CCI score, for each increase in CCI by 1 unit, the odds of 30-day mortality increased by 1.86 units (OR: 2.86, 95% CI: 1.05-7.77; p = 0.039) in our cohort (Tables 13-14).

**Table 13:** Binary logistic regression for risk factors of the occurrence of readmission within 1-year. *Significant at 0.05; **Significant at 0.01 GERD, Gastroesophageal reflux disease

Risk factors	Reference category	p-value	Odd ratio	95% CI for odd ratio
Nationality	Non-Bahraini	0.051	4.530	(0.99, 20.72)
Smoking status	Non-smoker	0.033*	3.273	(1.10, 9.76)
Melena	No	0.597	1.367	(0.43, 4.36)
GERD	No	0.259	2.231	(0.55, 9.00)
Rescope	No	<0.001**	10.911	(3.99, 29.83)

**Table 14:** Binary logistic regression for risk factors of the occurrence of 30-day mortality. *Significant at 0.05; **Significant at 0.01 CKD, Chronic kidney disease; CCI, Charlson Comorbidity Index

Risk factors	Reference category	p-value	Odd ratio	95% CI for odd ratio
Smoking status	Non-smoker	0.032*	0.028	(0, 0.73)
Organ damage	No	0.003**	0.002	(0, 0.13)
Hypertension	No	0.016*	3068.076	(4.45, 2114605.78)
Cardiac history	No	0.169	0.101	(0, 2.66)
Prior myocardial infarction	No	0.006**	849.693	(6.76, 106786.03)
Heart failure	No	0.182	45.374	(0.17, 12247.33)
Hemiplegia	No	0.058	77.460	(0.87, 6926.76)
Moderate/severe CKD	No	0.006**	320455.517	(36.04, 2849167704.23)
Warfarin	No	0.052	482.499	(0.94, 246951.83)
Abdominal pain	No	0.885	1.285	(0.04, 38.75)
Vomiting	No	0.061	13.381	(0.89, 201.53)
International normalized ratio	-	0.552	0.384	(0.02, 9.01)
Alkaline phosphatase (IU/L)	-	0.001**	1.057	(1.02, 1.09)
Creatinine (mmol/L)	-	0.008**	0.958	(0.93, 0.99)
CCI	-	0.039*	2.860	(1.05, 7.77)

## Discussion

UGIB remains one of the most common life-threatening emergencies [1]. In this study, we sought to investigate the epidemiology and clinical characteristics of UGIB in a Middle Eastern Bahraini cohort, where the literature is very limited. According to a recent systematic review, the overall mortality rate of UGIB ranges from 0.9 to 9.8 per 100,000 person-years, with a case-fatality rate estimated between 0.7% and 4.8%, depending on the underlying cause and the availability of resources [22]. The most common etiologies of UGIB, according to worldwide studies, are peptic ulcers and varices, followed by erosive gastritis, Mallory-Weiss tears, and vascular lesions [23-25]. However, the frequencies of the different etiologies show significant heterogeneity among populations, influenced by the prevalence of different predisposing factors [26].

The mean age of patients who presented with UGIB to our center was 56.7 ± 19.1 years, with 44.8% of these patients being over the age of 60. Similarly, a study from neighboring Riyadh, Saudi Arabia, reported an average age of 57.1 years. In comparison, a national audit conducted in the UK in 2007, which included 6,750 patients from 208 hospitals, found that the mean age was 68, with 63% of the population over 60. Additionally, comorbidities such as hypertension, diabetes mellitus, and ischemic heart disease are more common among patients in our study and perhaps the wider region, with the prevalence of patients with at least one comorbidity being 68% (Bahrain) and 88% (Saudi Arabia) vs. 46% (the UK) [27,28]. Thus, patients who suffer from UGIB in the Gulf region are typically younger and significantly more likely to have multiple comorbidities. The high prevalence of metabolic comorbidities developed earlier in the Middle Eastern population than in Western counterparts is most likely linked to genetic predisposition combined with high-calorie diets rich in sugar and fats [29,30]. As such, the role of dietary habits and early onset modifiable risk factors in exacerbating UGIB risk should be considered, especially as these factors seem to compound the effects of PUD and other gastrointestinal disorders in this demographic [31-33].

Non-gastrointestinal comorbidities have been established as predictors of poor prognosis in patients with UGIB, which our study corroborates [34]. Our study confirmed that non-gastrointestinal comorbidities are predictors of 30-day mortality in the population in Bahrain. For each increase in CCI by 1 unit, the odds of 30-day mortality increase by 1.86 units (186%) (p = 0.039) in our cohort. Therefore, irrespective of the severity of presentation, laboratory parameters, and need for blood products, the greater the number of comorbidities in patients with UGIB, the higher the risk of 30-day mortality. Moreover, there are isolated comorbidities that were associated with an increased risk of 30-day mortality: hypertension, prior MI, and moderate to severe CKD. Given the high prevalence of comorbidities, the patient population in Bahrain, as evidenced in this study, is at an increased risk of morbidity and mortality resulting from UGIB. This warrants increased vigilance and maintaining a lower threshold for escalating the treatment of UGIB in such patients.

CKD is an independent risk factor for GI bleeding. It is associated with an increased risk of gastritis, PUD, and angiodysplasia, and has worse outcomes than patients with normal renal function [35,36]. A study has found that the risk of mortality increases further with disease progression to end-stage renal disease (ESRD) [36]. The pathophysiology of UGIB in ESRD is multifactorial. Uremia in ESRD impairs platelet aggregation and adhesion by reducing platelet glycoprotein expression and inhibiting von Willebrand factor activity, leading to a uremic bleeding diathesis [37]. Furthermore, ESRD, as mentioned above, is associated with an increased prevalence of secondary angiodysplasia, particularly in the gastrointestinal tract, likely due to chronic vascular remodeling and ischemia [38]. The intermittent use of anticoagulants in dialysis patients further compounds the risk, creating a delicate balance between thrombosis and hemorrhage [39]. Our study corroborates these findings, showcasing that the risk of mortality in patients with moderate to severe CKD increases by more than 320,000 times (p = 0.006; 95% CI: 36.04-2849167704.23). These results suggest that prophylactic PPI doses may play a role in the prevention of UGIB exacerbation in patients with concurrent CKD, owing to the increased risk of mortality. This is further supported by several studies that have found that prophylactic low-dose PPIs can reduce the risk of UGIB in ESRD [40,41].

In addressing the elevated UGIB risk observed among patients with significant comorbidities, this study points to the potential benefit of integrated care pathways and multidisciplinary clinical models [17,42-44]. These can allow for collaborative management of patients with overlapping conditions such as CKD, cardiovascular disease, and diabetes, which we know exacerbate the risks of UGIB. These models can help coordinate care across specialties and reduce gaps in the management and monitoring of high-risk patients. Additionally, enhanced patient education and self-management programs would further support this model by allowing patients to recognize early UGIB symptoms and manage their risk factors effectively.

**Figure 1:**
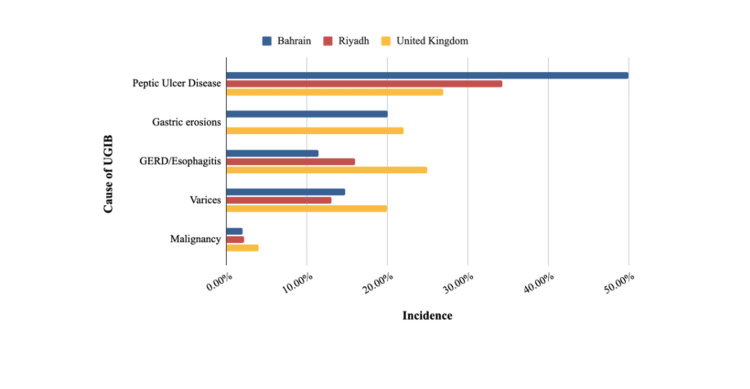
Incidence of the different causes of UGIB in Bahrain, Riyadh (2021), and the United Kingdom (2007). UGIB, Upper gastrointestinal bleeding; GERD, Gastroesophageal reflux disease

A comparison of the incidence of the different etiologies of UGIB in Bahrain (SMC), Riyadh, and the UK is demonstrated in Figure 1. In our cohort, PUD contributed to 42.5% of the total etiology of UGIB, with duodenal ulcers (37.7%) being the most prevalent finding, followed by gastric ulcers (13.6%). In comparison, PUD contributed to 34.3% of UGIB cases in Riyadh and 27% in the UK [44]. This data correlates with the prevalence of *H. pylori*; the higher prevalence of *H. pylori* (55.5%) in Bahrain compared to 35% in the UK could explain the higher number of PUD cases [45,46]. Moreover, according to a 2013 cohort study based in Taiwan, diabetes mellitus - which is highly prevalent in our population - may be an independent risk factor for PUD [47]. Additionally, a recent study reported that a high serum blood glucose level was associated with a rise in the incidence of acute UGIB in diabetic patients admitted with diabetic ketoacidosis (DKA) [48]. This further supports the potential relationship between diabetes and the higher rates of PUD contributing to UGIB in Bahrain.

As for variceal bleeding, there was a smaller margin of difference between the three studies in Bahrain (17.1%), Riyadh (13.1%), and the UK (20%) [26,27]. This may indicate that the risk factors for variceal bleeding are rather universal. Regarding mortality related to variceal bleeding, a study by Fallatah et al., based in Jeddah, Saudi Arabia, reported a mortality rate of 15.2% during the first episode of variceal bleeding [49]. Another study by Bilal et al., utilizing data from 22 states across the US, found that the 30-day mortality rate for patients admitted for esophageal variceal hemorrhage is 8.2% [50]. On the other hand, in our cohort, the readmission rate for patients admitted with variceal bleeding is as follows: 25% of patients admitted with esophageal varices, 12.5% of patients with fundal varices, and 33.3% of patients with both esophageal and fundal varices. Moreover, 12.5% of patients who suffered from fundal varices experienced 30-day mortality, and there was no mortality among patients with esophageal varices. However, there was no association between variceal bleeding (of any location) and one-year readmission or 30-day mortality.

Aspirin and NSAIDs were the most common medications taken by patients presenting with emergency UGIB. Chronic use of NSAIDs has been known to be associated with an increased likelihood of severe UGIB due to gastric and duodenal mucosal ulceration [51]. Nonetheless, the reported rate of NSAID use in our study was lower than in other cohorts, at 6.7%, compared to the 12% reported in the UK audit and the 13.6% reported in Riyadh [26,27]. A possible reason for such low reported usage is that NSAIDs, such as ibuprofen and diclofenac, are widely available in the country as over-the-counter medications, and many people consume them without a doctor’s prescription; as a result, these medications are not directly entered into the electronic medical system but rather depend on the documentation of the treating physician. Furthermore, this hinders the ability to properly examine the differences between PUD and variceal bleeding in our population. More accurate pharmacist-led documentation or patient self-reporting tools for over-the-counter NSAID use would result in more representative epidemiological data, improving our understanding of the true implications of medication use on UGIB in the country.

In our population, 12 patients (5.7%) were receiving warfarin as part of their medication regimens, six of whom did not survive beyond 30 days of their admission. Among all the medications being examined in our study, only warfarin usage was strongly associated with a mortality risk within 30 days of UGIB (p = 0.003). However, the sample size was small. Of note, a study by Majeed et al., based in Sweden, found that restarting vitamin K antagonists (VKAs) in cases of UGIB was associated with a reduced risk of thromboembolism and death, but with an increased risk of recurrent bleeding [52]. This risk decreased if VKAs were resumed after three weeks and reached a nadir at six weeks after the index gastrointestinal bleed. Thus, it may be worth investigating this further to improve the evidence base for guidelines on the initiation, suspension, and restarting of warfarin and other blood thinners in the context of UGIB.

With regard to clinical presentation in UGIB, we found that melena (62.1%) and symptoms of anemia (51.7%) were the most common, rather than hematemesis (23.7%). Hematemesis usually presents in severe and massive UGIB, such as ruptured esophageal or gastric varices [53]. In line with the literature, these findings were supported by the fact that most of the UGIB cases in this study suffered from inflammatory or erosive etiologies, such as duodenal ulcers, gastric ulcers, and gastroesophageal reflux disease (GERD), as opposed to varices. This can be explained by advanced liver disease being less common in the Gulf region [49], likely due to lower levels of alcohol consumption and chronic liver disease generally in the Eastern Mediterranean health region, compared to many other regions worldwide.

One of the interesting results of our study was the strong relationship between the need for a re-endoscopy during admission and the likelihood of readmission within one year. In our cohort, we found that patients who required a re-endoscopy during their admission were around 10 times more likely to be readmitted within one year than patients who did not require a re-endoscopy (p < 0.001). We hypothesize that this may be attributed to the fact that these patients may require a re-endoscopy due to the severity of UGIB, lack of resolution of bleeding, additional therapeutic interventions, uncertainty of diagnosis, or lack of findings on EGD. The ambiguity in diagnosing UGIB or identifying the cause of UGIB may contribute to suboptimal therapy, resulting in readmission in such patients. Data are insufficient to explore this hypothesis, and there is an area for future research in this population of patients. Possible areas of improvement could include exploring the utility of second-look endoscopies in cases where initial resolution is incomplete or where the patient presents with higher Forrest classifications of bleeding, ensuring more robust initial management [54,55]. Likewise, for complex cases requiring multiple interventions, establishing a follow-up protocol to monitor recurrence risk within the first few weeks post-discharge might help mitigate the readmission rate [56].

Our study had an average mortality rate of 15.6% and an average readmission rate of 14.2%. On the other hand, in Riyadh, a smaller mortality rate of 4.4% was reported for patients with UGIB [7]. In comparison, a study based in the US found that the total 30-day mortality rate was 10.4% [57]. Similar numbers can be found in the UK, with an in-hospital mortality of 10% [27]. Nevertheless, the higher mortality rate, despite similar baseline demographics and burden of comorbidities in Riyadh, supports the conclusion that there is a need for further studies and monitoring of the local UGIB cohort with regard to accurate diagnosis and optimization of management, in addition to identifying potential contributors to the high mortality rate that is unique to UGIB patients in Bahrain. While the causes behind this disparity are not fully clear, it may be partially attributed to variations in healthcare access, as well as differences in post-UGIB management and follow-up protocols. Given the high proportion of patients admitted through the ED, it is plausible that many cases are not detected until they are more severe, resulting in heightened mortality. This would restate the necessity of primary and preventive care services to identify and manage risks before they escalate to emergency UGIB events, as well as the importance of timely intervention to improve outcomes [58]. Automated scoring integrated within electronic health records could facilitate efficient triage and enhanced access to early endoscopy [59], as early endoscopic intervention is associated with improved outcomes. Establishing fast-track endoscopy units in high-volume EDs, including prepackaged UGIB kits, and improved ED staff awareness of UGIB may all contribute to improved patient outcomes, and it would be valuable to conduct two-cycle audits on.

Strengths and limitations

The study demonstrated several strengths and limitations that warrant discussion. Among its strengths, it utilized a comprehensive dataset of 212 patients, allowing for robust statistical analysis and significant insights. The regional relevance of the study is notable, as it provides valuable data on UGIB in Bahrain, a region with limited prior research. Methodological rigor was ensured through the use of validated risk stratification tools, such as the GBS and Rockall scores, and a focus on key outcomes, including 30-day mortality and one-year readmission rates, offered a holistic perspective. Additionally, the identification of risk factors like CKD and cerebrovascular accidents provides actionable insights for clinical practice. Ethical considerations were also upheld through patient anonymity and secure data handling. However, the study faced some limitations. Its single-center scope may limit the generalizability of findings to other regions or healthcare systems. The retrospective design, while valuable, introduces potential biases and limits causal inference. Additionally, underreporting of over-the-counter NSAID use and small sample sizes in certain subgroups, such as those receiving warfarin or rescope procedures, constrain the reliability of specific analyses. Furthermore, lifestyle and dietary factors were not included, and longer-term outcomes beyond one-year readmissions were not explored. These limitations suggest opportunities for future research to build on the study’s findings and broaden its applicability.

## Conclusions

UGIB is a widely prevalent, life-threatening emergency worldwide. The common risk factors for UGIB in Bahrain include prior bleeding, the use of NSAIDs and warfarin, and cardiovascular disease. The most common causes of UGIB in Bahrain include PUD (predominantly duodenal), gastric erosions, and variceal bleeding. The most common symptoms at presentation include melena, coffee-ground vomiting, and symptoms of anemia. Presentations involving tachycardia, low blood pressure, anemia, and low oxygen saturation were not uncommon. PRBCs were administered in approximately one in two patients with UGIB in Bahrain, while FFP and platelet transfusions were reserved for severe cases. The mortality rate of UGIB in Bahrain is slightly higher than average, at 15.6%, compared to countries in the region, the UK, and the US, signaling potential gaps in early diagnosis, comprehensive management, and follow-up care, and maybe a reflection of a more complex patient population, both of which require further study. The strong association between the need for a rescope during hospitalization and higher readmission rates within one year underscores the importance of optimizing initial therapeutic interventions and follow-up protocols.

This research offers valuable insights into the clinical landscape of UGIB in Bahrain, contributing to the limited regional literature on the topic. The findings advocate for targeted public health strategies, including enhanced preventive measures, such as early screening for high-risk groups, better documentation of over-the-counter NSAID usage, and education on medication risks. Future research should focus on longitudinal studies that explore the impact of integrated care pathways and advanced therapeutic protocols on patient outcomes. By situating UGIB outcomes within the broader epidemiological and healthcare context, this study lays the groundwork for improving management strategies, reducing mortality rates, and ultimately enhancing the quality of care for UGIB patients in Bahrain and beyond. Addressing the highlighted gaps through systematic and multidisciplinary approaches could significantly mitigate the burden of this life-threatening emergency.
